# Ring of Fire: The Case of Mistaken Identity

**DOI:** 10.7759/cureus.29452

**Published:** 2022-09-22

**Authors:** Clates P Adams, Andrew Zabel, Vanessa Hannick

**Affiliations:** 1 Medicine, William Beaumont Army Medical Center, Fort Bliss, USA; 2 Emergency Medicine, Carl R. Darnall Army Medical Center, Fort Hood, USA

**Keywords:** heterotopic pregnancy, pregnancy, ring of fire, colored flow doppler ultrasound, corpus luteal cyst, heterotopic pregnancy (hp), bleeding in pregnancy

## Abstract

This case investigates the difference between heterotopic pregnancy vs. intrauterine pregnancy, a topic that more emergency medicine physicians will face as the rates of assisted reproductive technologies increase. Throughout the case, the pathognomonic "Ring of Fire" is discussed, as is seen on the transvaginal ultrasound, and its reliability is assessed.

## Introduction

Heterotopic pregnancy is the presence of simultaneous pregnancies at two different implantation sites. Most commonly, these sites are a combination of intrauterine and extrauterine pregnancies [1]. In the setting of a true intrauterine pregnancy (IUP), signs suggestive of heterotopic pregnancy include complex adnexal masses or fluid in the pelvis. Additionally, color flow Doppler does not differentiate an ovarian pregnancy from a corpus luteal cyst, since the "Ring of Fire" appearance can be seen for either case and should no longer be considered pathognomonic for heterotopic pregnancy.

## Case presentation

A 30-year-old pregnant female with a medical history of infertility of unknown cause presented at seven weeks and three days of gestation (confirmed by ultrasound and quantitative beta-human chorionic gonadotropin (HCG) of 40,000 mIU/mL, at an outside emergency department one week prior), with several days of scant vaginal bleeding and mild pelvic pain. A review of the cardiac, pulmonary, gastrointestinal, and genitourinary systems was otherwise normal. On physical exam, vital signs were clinically unremarkable. The patient was well-appearing and in no apparent distress, and her abdomen was soft and gravid with mild tenderness in the lower abdomen, bilaterally. Her cardiovascular exam demonstrated a regular rate and rhythm, with no murmur, gallop, or rub. Pulses were 3+ and equal in all extremities. A pulmonary exam showed non-labored breathing and lung fields were clear to auscultation bilaterally. Her quantitative beta-HCG was 120,000.

**Figure 1:**
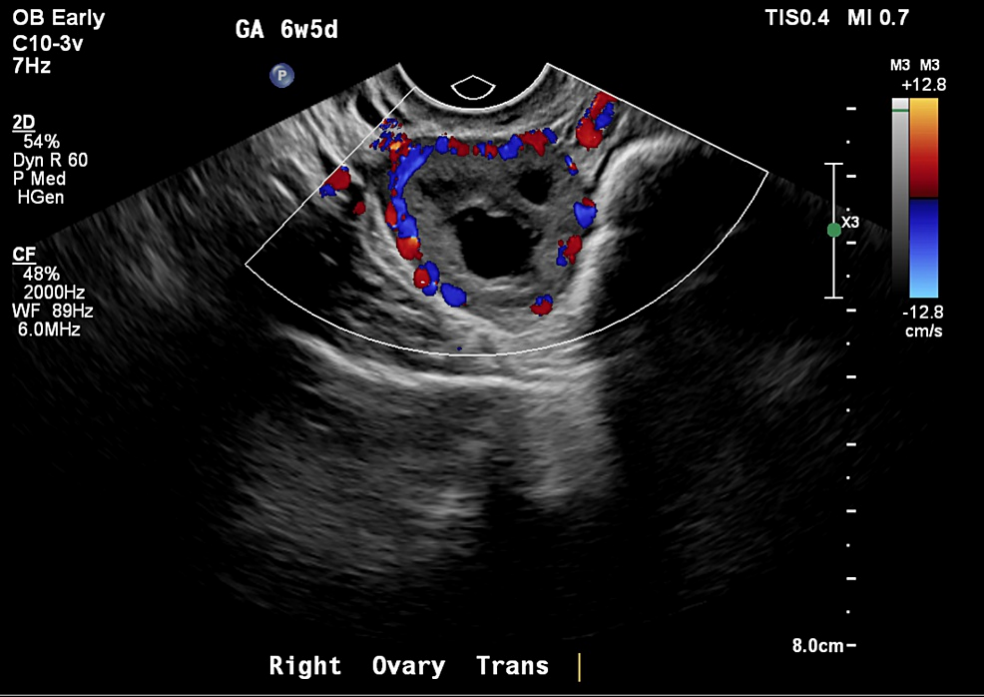
"Ring of Fire" on ultrasound

Transvaginal color flow Doppler ultrasound was ordered and revealed the pathognomonic "Ring of Fire" (Figure 1). After obtaining the Doppler ultrasound image, the obstetrician on-call was consulted, and they favored the diagnosis of corpus luteal cyst over heterotopic pregnancy. The patient was re-evaluated by obstetrics the next morning, and several other physicians agreed. This case was not a heterotopic pregnancy and the patient went on to have a normal pregnancy.

## Discussion

Heterotopic pregnancies have been diagnosed from five to 34 weeks of gestation. A total of 70% are diagnosed between five and eight weeks, 20% between nine and 10 weeks, and only 10% after the 11th week [2]. The historical incidence is 1/30,000 [3,4]. However, since the relative common use of assisted reproduction techniques, incidences have increased to 1/3,900 patients [5], and in those who receive in vitro fertilization (IVF), rates are quoted as 1/100 [6]. Interestingly, color flow Doppler does not differentiate an ovarian pregnancy from a corpus luteal cyst, since a “Ring of Fire” appearance can be seen for either according to the literature [7]. Although not performed in this case, anti-Müllerian hormone (AMH) could have been used to differentiate between the diagnoses.

The standard of treatment for an ectopic pregnancy, in the setting of a coexistent intrauterine pregnancy, is a laparoscopic salpingectomy and/or oophorectomy, to permit the intrauterine pregnancy to progress normally. Therefore, being able to differentiate between an ectopic and corpus luteum cyst will vastly alter the care and potential complications a patient could encounter.

## Conclusions

There is a need for physicians to be alert in cases of pregnancy after IVF and those with concerning findings on transvaginal ultrasound. Confirming an IUP clinically, or by ultrasound, does not exclude the coexistence of ectopic pregnancy. It should be kept in the differential, suspected, and confirmed with AMH, in any patient presenting with abdominal pain or bleeding during pregnancy. The use of assisted reproduction techniques is increasing the likelihood of seeing a heterotopic pregnancy. A physician's failure to keep heterotopic pregnancy on the differential, after visualizing an IUP, can lead to a missed diagnosis and a catastrophic outcome for the mother.
